# Episode-wide Maudsley staging in treatment-resistant depression: a longitudinal tertiary-care comparison of dimensional and categorical outcomes

**DOI:** 10.3389/fpsyt.2026.1769246

**Published:** 2026-02-11

**Authors:** Anastasia Antoniou, Sofia Pappa, Dimitrios Dikeos, Nikolaos Smyrnis, Julien Mendlevicz, Alessandro Serretti, Anthony Cleare, Panagiotis Ferentinos

**Affiliations:** 1 2nd Department of Psychiatry, National and Kapodistrian University of Athens, “Attikon” University General Hospital, Athens, Greece; 2 Department of Brain Sciences, Faculty of Medicine, Imperial College London, London, United Kingdom; 3 West London NHS Trust, London, United Kingdom; 4 1st Department of Psychiatry, National and Kapodistrian University of Athens, Eginition Hospital, Athens, Greece; 5 Université Libre de Bruxelles, Brussels, Belgium; 6 Department of Medicine and Surgery, Kore University of Enna, Enna, Italy; 7 Oasi Research Institute-IRCCS, Troina, Italy; 8 Institute of Psychiatry, Psychology & Neuroscience, King’s College London, London, United Kingdom; 9 South London and Maudsley NHS Foundation Trust, London, United Kingdom

**Keywords:** depression, major depressive disorder (MDD), Maudsley staging method (MSM), remission, treatment-resistant depression (TRD)

## Abstract

**Background:**

Unlike typical categorical definitions of treatment-resistant depression (TRD) based on antidepressant (AD) trial failures, the Maudsley Staging Method (MSM) is a dimensional measure of resistance also rating episode duration, baseline depression severity and failure of augmentation strategies and ECT. However, MSM has not previously been used longitudinally across the full depressive episode, from episode onset to remission. We applied episode-wide MSM (EW-MSM) in patients with Major Depressive Disorder naturalistically followed to remission in a tertiary-care setting and categorized as 1^st^ and 2^nd^ AD trial remitters, TRD remitters and TRD non-remitters (categorical outcomes). The study aimed to investigate clinicodemographic and treatment-related correlates of EW-MSM and categorical outcomes, comparatively assess their predictive value for depression improvement, and explore the discriminative utility of EW-MSM across categorical outcomes.

**Methods:**

We recruited 267 patients. EW-MSM was scored at remission (MADRS ≤ 9 in two consecutive visits), if achieved. Associations of clinicodemographic and treatment-related characteristics with EW-MSM and categorical outcomes were explored in multivariate models. Their comparative predictive value for depression improvement was tested in hierarchical linear regressions. ROC curves assessed EW-MSM discriminative utility across categorical outcomes.

**Results:**

Analysis focused on 233 remitters (105 1^st^ AD trial remitters, 62 2^nd^ AD trial remitters and 66 TRD remitters). Both EW-MSM and categorical outcomes were associated with baseline severity, obsessive-compulsive disorder comorbidity, episode duration, number of ADs or AD combination. EW-MSM was additionally associated with psychotic features and use of augmentation strategies. EW-MSM was more strongly associated with baseline severity than categorical outcomes (R^2^ = 0.24 vs. 0.14) and consequently predicted depression improvement to remission more efficiently in hierarchical regressions (ΔR^2^ = 0.13 vs. 0.05). EW-MSM discriminated well TRD remitters from 1^st^ AD trial remitters (AUC = 0.91, 95% CI = 0.86-0.95) or from early remitters combined (AUC = 0.865, 95% CI = 0.81-0.92) but not among adjacent categories.

**Limitations:**

Single, tertiary-care setting, unavailability of ECT/esketamine and exclusion of patients on psychotherapy (not rated by MSM) limit study generalizability.

**Conclusions:**

EW-MSM reflects clinical and treatment intensity aspects of resistance in depression more strongly and comprehensively than categorical outcomes. Therefore, it offers precision for staging and could be used to more efficiently investigate clinical, biological and psychological correlates of resistance.

## 1 Introduction

Major Depressive Disorder (MDD) is among the most prevalent and disabling psychiatric conditions worldwide (1). Despite numerous available evidence-based pharmacological treatments, a substantial proportion of patients do not achieve satisfactory outcomes and develop treatment-resistant depression (TRD) (2, 3). Compared to MDD, TRD is associated with higher rates of psychiatric and physical comorbidities, greater functional impairment, increased likelihood of hospitalization, longer hospital stays, higher healthcare costs, and an elevated risk of suicidality (4–11).

Despite its clinical relevance, a consensual operational definition and diagnostic criteria for TRD are lacking (12–14). There is no solid evidence validating any meaningful cut-off based on the number of treatment failures for defining a binary TRD phenotype and a spectrum of resistance seems more plausible (15, 16). Yet, according to the European Medicines Agency (EMA), TRD is considered when treatment with at least two different antidepressant agents (of the same or a different class) of adequate dosage and duration shows lack of clinically meaningful improvement (17). Based on this statement, TRD is frequently defined as failure to respond to two adequate antidepressant (AD) trials. Using this definition, approximately 30% of patients with MDD develop TRD (2, 6).

However, not all responders finally achieve the ultimate therapeutic goal, which is clinical remission, i.e. near-absence of depressive symptoms (18). Therefore, if TRD is defined as lack of clinical remission rather than lack of response after two AD trials, its estimated prevalence rises considerably. For example, in the STAR*D study (19), after the first two trials around 45% of patients cumulatively failed to reach remission (20). Full restoration of psychosocial and cognitive functioning (functional recovery), recently recognized as the optimal goal of treatment, typically takes much longer to achieve, and its measurement varies widely across settings (21). Therefore, clinical remission, signifying the end of the depressive episode, appears as a reasonable preferable clinical anchor on which definition of resistance should be based (22).

Apart from binary definitions of TRD, such as lack of response or remission after two AD trials, which are often used in clinical practice as they are simpler and adopted by regulation authorities, dimensional and staging models have also been proposed to describe multiple dimensions of treatment resistance or gradations in its severity, respectively, rather than simply presence or absence of treatment resistance (13, 14, 23). Staging frameworks include the Thase and Rush model (24), which describes five stages of resistance depending on the pharmacological AD classes sequentially failed and assuming a hierarchy of ADs, and the European Staging Model (ESM), proposed by the Group for the Study of Resistant Depression (GSRD), which describes three main stages of resistance depending on the number and duration of failed AD trials (25, 26).

The Maudsley Staging Method (MSM) is both a dimensional and a staging tool to assess treatment resistance in MDD (27). In addition to AD failures, MSM also rates failure of any augmentation strategies and electroconvulsive therapy (ECT). Unlike earlier staging models, MSM uses lack of remission rather than lack of response to define treatment failures (22). Moreover, it bases assessment of resistance not only on treatment failures but also two additional dimensions, duration of current episode and baseline depression severity. The total MSM score also allows for the classification of patients into mild, moderate or severe stages of resistance. The model has been validated in multiple studies, demonstrating its predictive value for poor prospective clinical outcomes, including increased functional impairment, time spent with depression and lower chance of remission (22, 28–32). By integrating both treatment intensity and clinical burden (depression severity and episode duration), this remission-based dimensional staging model provides additional benefits for treatment resistance quantification both in research and clinical settings.

Despite growing interest in TRD, there is a notable lack of longitudinal, real-world studies following up TRD patients to remission in naturalistic settings. Most existing studies prioritize treatment response as the primary outcome and often have limited follow-up durations, rarely exceeding two years. Regarding MSM, it has been applied only at random snapshots, e.g. at hospital discharge, to predict short- or long-term outcomes. To date, no studies have used MSM to quantify the full continuum of treatment resistance across the Major Depressive Episode (MDE) ending at remission, i.e. episode-wide. Capturing the total burden of treatment failures and time elapsing from episode onset to remission as well as baseline clinical severity in a dimensional framework, such as an episode-wide version of MSM, produces an accurate, continuous index of resistance observed over the whole episode. This index is not an estimate or prediction but a *post-hoc* record of all treatment efforts required and time needed to reach remission given the initial severity. Therefore, it can serve as a gold standard to compare with yet non-validated categorical classifications, such as remitters, non-remitters or TRD. Furthermore, it might be tested for correlation with candidate biological, clinical or psychological predictors.

This prospective observational study included adult patients with a moderate to severe MDE in the context of MDD who were naturalistically followed up at a specialized tertiary-care center aiming at remission. It was part of the ATLADIS (AThens Longitudinal Affective DIsorders Study) prospective study also including patients with bipolar disorder (33, 34). Using the GSRD/ESM categorical TRD definition but adopting a remission-based framework, patients were categorized as 1^st^ AD trial remitters, 2^nd^ AD trial remitters, TRD remitters and TRD non-remitters (categorical outcomes). Episode-wide MSM (EW-MSM) was rated at the end of the MDE (on remission, if achieved) to produce a continuous score of resistance of the MDE from its beginning to its end. The study aimed to:

1. Examine the association of sociodemographic and clinical characteristics, baseline mood severity, and current MDE treatment parameters with categorical outcomes and EW-MSM scores.
2. Compare the predictive value of EW-MSM versus categorical outcomes for improvement in depression severity.
3. Evaluate the discriminative utility of EW-MSM across categorical outcomes.

## 2 Methods

### 2.1 Participants

Included patients were diagnosed with MDD as the primary psychiatric diagnosis and suffered from a current MDE, according to Diagnostic and Statistical Manual of Mental Disorders (DSM)-5 criteria, as assessed with the Structured Clinical Interview for DSM-5 Disorders - Clinician Version (SCID-5-CV) by an experienced psychiatrist (35). An at least moderate severity of the MDE was required for inclusion as documented by a Montgomery Åsberg Depression Rating Scale (MADRS) (36) score ≥20 (37). Patients were recruited from the specialized tertiary-care Affective Disorders and Suicidality outpatient clinic at the 2^nd^ Department of Psychiatry, Medical School of National and Kapodistrian University of Athens, located in Attikon University Hospital, Athens, Greece between January 2014 and June 2023. Follow-up at the clinic started either before or after the onset of the current MDE. Patients with neurocognitive disorders (delirium, dementia, traumatic brain injury, etc.), intellectual disability (IQ<70), lack of fluency in Greek, severe comorbid psychiatric disorders (alcohol and substance abuse during the last 6 months, severe personality disorder) and severe uncontrolled medical conditions (e.g. neoplasms) were excluded. As MSM does not rate 0 ADs, only patients treated with at least one AD at an adequate dose for a minimum of four weeks were included. As MSM does not include psychotherapy, patients who received psychotherapy for the current episode (independent of whether they did so in previous episodes) were also excluded.

The study protocol was approved by the local Ethics Committee and all procedures adhered to the principles of the Declaration of Helsinki. Written informed consent was obtained from all participants.

### 2.2 Treatment procedures

Patients were naturalistically followed up every 1–3 months, or more often when clinically justified (suicidality, poor AD tolerability, hypomanic switch). Monotherapy, combination therapy and augmentation strategies were freely allowed based on clinical judgement and established guidelines, aiming to achieve remission. ECT, repetitive transcranial magnetic stimulation (rTMS) or intranasal esketamine were not available in our setting. ADs were recorded only if administered for at least four weeks on at least the minimum recommended dose(s). An AD trial was defined as a period of treatment with ADs, one or more (AD combination trial), of at least minimum dosage and duration, and any augmentation agents. Augmentation strategies, typically involving the addition of antipsychotics and mood stabilizers, were recorded if lasting at least four weeks and were considered part of an AD trial rather than a separate trial. The addition of benzodiazepines or hypnotics was not classified as augmentation.

Criteria of unsatisfactory outcome triggering decisions to change treatment (dose optimization, augmentation, combination or switching) consistently included:

- No response (<25% reduction in MADRS score) after ≥4 weeks of adequate treatment.
- Partial response (25–49% reduction in MADRS score) after ≥6 weeks of adequate treatment.
- Lack of remission (MADRS ≥10) after ≥12 weeks of adequate treatment.
- Earlier treatment modifications in cases of poor tolerability.

However, treatment decisions were individualized, with no predefined criteria guiding the selection of specific medications or treatment adjustments.

All administered treatments after the onset of the current MDE were thoroughly recorded for the whole episode and also specifically extracted for the first and last AD trials. Current MDE treatment characteristics (number of ADs and AD trials used, AD combinations and augmentation strategies employed) were extracted. The maximum AD doses received for at least 4 weeks episode-wise and in first and last AD trials were also extracted. AD doses were also converted to fluoxetine dose equivalents (38, 39).

The study ended in June 2024. For any individual patient, participation in the study was completed when remission was reached or, before remission was reached, when the study ended or the patient dropped out. The last two options were applicable only for TRD patients, i.e., those with 3 or more AD trials. Hence, patients who ceased follow-up early, after the 1^st^ or 2^nd^ AD trial without achieving remission were excluded from the analysis. The total duration of current MDE was determined from episode onset to remission, study end if earlier or drop out.

### 2.3 Clinical assessments

Clinicodemographic (sociodemographic and clinical) data were obtained through semi-structured clinical interviews conducted by an experienced psychiatrist or clinical psychologist. Follow-up of the current MDE always started after its onset, which was determined based on patient self-reports, caregivers’ information and medical records after assessing their reliability; conflicts were resolved by contacting patients’ former therapists. Baseline MADRS and Young Mania Rating Scale (YMRS) (40) scores were retrospectively assessed (R-MADRS, R-YMRS) to evaluate symptom severity at episode onset using the same procedures. MADRS was also rated when follow-up of the current MDE started (First evaluation MADRS). Mood state monitoring was primarily based on the Clinical Global Impressions Scale – Severity (CGI-S) (41), with optional MADRS assessments, when justified by clinical circumstances, and YMRS assessments, when hypomania was suspected (YMRS ≥ 12). When CGI-S reached 1 or 2, remission was tested using MADRS (≤9) and YMRS (≤6) cut-offs in two consecutive visits (42, 43). Δ-MADRS was calculated as baseline MADRS (R-MADRS) – remission MADRS.

MSM (27) assesses three core dimensions of treatment resistance: duration of current episode (acute, sub-acute or chronic; 1–3 points), depressive symptom severity (subsyndromal, mild, moderate, severe without or with psychosis; 1–5 points) and the extent of treatment failure (1–7 points), which includes the number of AD failures (1–5 points), as well as whether augmentation strategies and ECT were used (1 point each if used). Scoring of the ECT item was not used in this study as ECT was not available in our setting. Apart from the scores of the three dimensions, the total MSM score, ranging from 3 to 15, is a single, continuous, dimensional measure of resistance, also allowing for the classification of patients into mild (scores 3-6), moderate (scores 7-10) or severe (scores 11-15) stages of resistance. In our study, EW-MSM was scored at the end of the episode, upon remission. Of note, scoring was based on the total number of ADs received during the episode, rather than treatment failures alone, as previously applied (29).

Furthermore, categorical outcomes were defined after modifying the GSRD categorical definition, distinguishing participants into 1^st^ AD trial remitters, 2^nd^ AD trial remitters, TRD remitters (after at least three AD trials) and TRD non-remitters (26).

### 2.4 Statistical analyses

Descriptive statistics were calculated for all variables in the total sample and across categorical outcomes. Normality was assessed using Shapiro – Wilk test. Univariate comparisons between groups were performed using Kruskal-Wallis, Pearson’s chi-square or Fisher’s exact test as appropriate. Univariate linear regressions were also conducted for EW-MSM total score to examine its associations with clinicodemographic characteristics, baseline mood severity and current MDE treatment characteristics.

Variables with p<0.1 in univariate comparisons were included in multivariate multinomial logistic regression models, assessing categorical outcomes in relation to clinicodemographic characteristics and baseline mood severity as well as current MDE treatment characteristics. Additionally, multiple linear regression models were constructed to examine the relationship between EW-MSM total score and the same sets of predictors, incorporating variables identified in univariate regressions (p<0.1). The variance explained by final multivariate models was estimated by R^2^ in linear regression models and by Cox-Snell pseudo-R^2^ in logistic regression models.

Hierarchical linear regression analyses were conducted to assess the added explanatory value of EW-MSM total score versus categorical outcomes while predicting Δ-MADRS, adjusting for sex and age. Inference was based on model comparisons.

The potential of EW-MSM in discriminating between categorical outcomes was investigated in Receiver Operating Characteristic (ROC) curves analyses, using categorical outcomes as reference variables and EW-MSM total score as classifier. The areas under the curves (AUCs) and their 95% CI were computed.

All statistical analyses were performed using STATA 14.0 (44).

## 3 Results

### 3.1 Study flow

**Figure 1:**
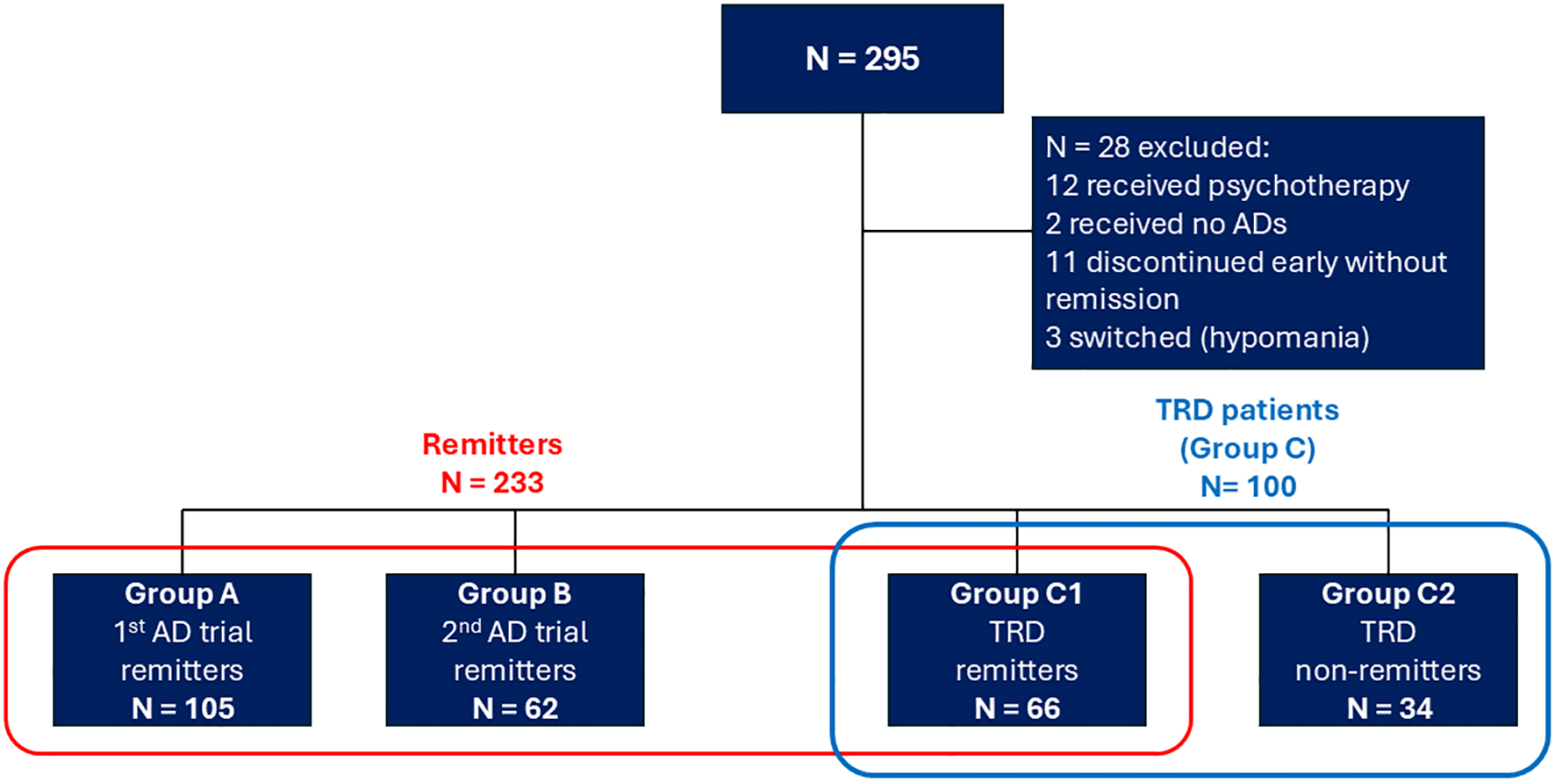
Overall participant flow. AD, Antidepressant; TRD, Treatment-resistant Depression.

No patients refused to participate. A total of 295 participants were initially enrolled. Twelve participants received adjunctive psychotherapy during the current episode and were hence excluded. Two additional patients were excluded as they received only quetiapine but no AD during the current episode. Furthermore, 11 individuals discontinued follow-up after the 1^st^ or 2^nd^ AD trial before achieving remission, therefore they could not be classified into any categorical outcome. Three individuals experienced a hypomanic episode (‘switch’) during AD treatment, necessitating a revision of their diagnosis. All 14 aforementioned individuals were excluded from the study, leaving 267 eligible participants (**Figure 1**). Patients on psychotherapy were not significantly different from the finally eligible participants on gender (*p* = 0.85), age (*p* = 0.38), educational attainment (*p* = 0.70) and R-MADRS (*p* = 0.31). At study end, no TRD patient had dropped out and 233 out of 267 (87.3%) patients (all 167 non-TRD and 66 TRD patients) had achieved remission. Of note, 105 participants (39.3%) were also included in the GSRD TRD-III (45) and TRD-IV (46) studies.

The primary analyses focused on the 233 participants who achieved remission following one (group A, 1^st^ AD trial remitters: N = 105, 45.1%), two (group B, 2^nd^ AD trial remitters: N = 62, 26.6%) and three or more AD trials (group C1, TRD remitters: N = 66, 28.3%). Thirty-four TRD participants did not reach remission by the end of the study (group C2, TRD non-remitters). TRD remitters (C1) and TRD non-remitters (C2) did not significantly differ on clinicodemographic characteristics, baseline mood severity (R-MADRS, R-YMRS) and most current MDE treatment characteristics (**Supplementary Tables 1**, **2**). The two groups differed, however, in that TRD non-remitters received treatment for longer (*p* = 0.004) but had a lower likelihood of having received AD combination treatment (*p* = 0.021).

### 3.2 Descriptives of the total remitters sample (N = 233)

Mean age of remitters (N = 233) was 52.3 ± 12.5 years and 62.7% were females. Among them, 73% suffered from recurrent MDD, while 63.9% and 23.6% exhibited lifetime melancholic and psychotic features, respectively. Furthermore, 30.9% had experienced a comorbid anxiety disorder and 51.5% had lifetime suicide attempts (**Supplementary Table 3**). Mean R-MADRS was 40.6 ± 5.7, mean remission MADRS was 5.5 ± 2.5 and mean Δ-MADRS was 35.1 ± 5.8 (**Table 1**, **Supplementary Table 3**). Median current MDE duration was 7 (range 2-120) months (**Table 2**). In those starting follow-up at our clinic after current MDE onset (N = 154, 66.1%), median time of initiation of follow-up of the current MDE was 3 (range 1-96) months after current MDE onset, while in those already followed up at our clinic before current MDE onset (N = 79, 33.9%), follow-up of the current MDE started one month (N = 39) or earlier after current MDE onset. Finally, 82% of remitters were hospitalized during their current MDE.

**Table 1:** Descriptive statistics and univariate analyses of clinicodemographic data and depression severity ratings in remitters (N = 233).

Variables	Group A 1^st^ AD trial remitters N=105	Group B 2^nd^ AD trial remitters N=62	Group C1TRD remitters N=66	All remitters N=233	Comparison between groups A, B, C1		EW-MSM on predictors*	
									Statistic	*P*-value	B	*P*-value
Clinicodemographic characteristics								
Age	51.37 ± 12.75	54.23 ± 11.48	51.95 ± 12.98	52.3 ± 12.5	2.712^1^	0.258	0.007	0.336
Sex (female)	69 (65.7%)	33 (53.2%)	44 (66.7%)	146 (62.7%)	3.230^2^	0.199	0.024	0.901
Number of hospitalizations (lifetime)	1 (1, 2)	1 (1, 2)	1 (1, 2)	1 (1, 2)	3.673^1^	0.159	0.121	**0.037**
Psychotic features (lifetime)	27 (25.7%)	13 (21%)	15 (22.7%)	55 (23.6%)	0.526^2^	0.769	0.996	**<0.001**
Current psychotic features	21 (20%)	12 (19.4%)	12 (18.2%)	45 (19.3%)	0.086^2^	0.958	1.226	**<0.001**
OCD (lifetime)	5 (4.8%)	8 (12.9%)	9 (13.6%)	22 (9.4%)	4.916^2^	0.086	0.579	0.065
First-degree relative with MDD	22 (21%)	21 (33.9%)	23 (35.4%)**	66 (28.4%)**	5.330^2^	0.070	0.291	0.155
Depression severity ratings: baseline/remission								
R-MADRS score	38.3 ± 5.23	42.63 ± 5.39	42.44 ± 5.4	40.62 ± 5.7	32.013^1^	**<0.001** **(B=C1>A)**	0.121	**<0.001**
First evaluation MADRS score	37.4 ± 5	41.73 ± 5.18	41.24 ± 5.05	39.64 ± 5.44	33.772^1^	**<0.001** **(B=C1>A)**	0.125	**<0.001**
Remission MADRS score	4.91 ± 2.37	5.35 ± 2.5	6.7 ± 2.36	5.54 ± 2.51	22.182^1^	**<0.001** **(C1>A=B)**	**-**	**-**
Δ-MADRS score	33.38 ± 5.19	37.27 ± 5.86	35.74 ± 5.98	35.09 ± 5.82	20.748^1^	**<0.001** **(B=C1>A)**	–	–

Displayed are mean ± SD or median (25^th^, 75^th^ percentiles) or N (%) in first 4 columns.

EW-MSM, Episode-wide Maudsley Staging Method; MADRS, Montgomery Åsberg Depression Rating Scale; MDD, Major Depressive Disorder; OCD, Obsessive Compulsive Disorder; R-MADRS, Retrospective baseline MADRS; TRD, Treatment Resistant Depression; Δ-MADRS, Retrospective baseline MADRS – Remission MADRS.

^1^Kruskall-Wallis test, ^2^Chi-square test, ^3^Fisher-Freeman-Halton exact test.

*Univariate linear regressions of EW-MSM total score (dependent variable) on various predictors, ** Missing data, total N = 232.

Bold p<0.05.

Apart from age and sex, only variables with p<0.1 in any analysis are displayed.

**Table 2:** Descriptive statistics and univariate analyses of current MDE treatment characteristics in remitters (N = 233).

Current MDE treatment characteristics	Group A 1^st^ AD trial remitters N=105	Group B 2^nd^ AD trial remitters N=62	Group C TRD remitters N=66	All remitters N=233	Comparison between groups A, B, C1		EW-MSM on predictors*	
									Statistic	*P*-value	B	*P*-value
Current MDE duration (months)	5 (4, 8)	8 (5.75, 10)	13 (8, 21)	7 (5, 11)	67.421^1^	**<0.001** **(C1>B>A)**	0.062	**<0.001**
Total number of ADs received	1 (1, 1)	2 (2, 3)	4 (3, 4)	2 (1, 3)	180.800^1^	**<0.001** **(C1>B>A)**	0.714	**< 0.001**
Total number of AD trials	1 (1, 1)	2 (2, 2)	3 (3, 4)	2 (1, 3)	227.656^1^	**<0.001** **(C1>B>A)**	0.757	**<0.001**
AD combination in any trial	21 (20%)	38 (61.3%)	56 (84.8%)	115 (49.4%)	72.995^2^	**<0.001** **(C1>B>A)**	1.217	**<0.001**
Fluoxetine dose equivalents for all ADs across MDE, mg/day	51.63 ± 24.22	93.25 ± 37.32	147.5 ± 59.83	89.86 ± 56.94	130.565^1^	**<0.001** **(C1>B>A)**	0.017	**<0.001**
Augmentation in any trial	79 (75.2%)	54 (87.1%)	61 (92.4%)	194 (83.3%)	9.480^2^	**0.009** **(C1>A)**	2.128	**<0.001**
AD combination in first trial	21 (20%)	17 (27.4%)	10 (15.2%)	48 (20.6%)	2.984^2^	0.225	0.179	0.432
AD combination in last AD trial	21 (20%)	32 (51.6%)	36 (54.5%)	89 (38.2%)	26.928^2^	**<0.001** **(B=C1>A)**	0.738	**<0.001**
Fluoxetine dose equivalents for ADs in last trial, mg/day	51.63 ± 24.22	59.68 ± 31.1	67.35 ± 29.7	58.22 ± 28.43	15.432^1^	**<0.001** **(C1>A)**	0.013	**<0.001**
Augmentation in last trial	79 (75.2%)	49 (79%)	57 (86.4%)	185 (79.4%)	3.074^2^	0.215	1.632	**<0.001**

Displayed are mean ± SD or median (25th, 75th percentiles) or N (%) in first 4 columns.

AD, Antidepressants; EW-MSM, Episode-wide Maudsley Staging Method; MDE, Major Depressive Episode; TRD, Treatment Resistant Depression.

^1^Kruskall-Wallis test, ^2^Chi-square test.

*Univariate linear regressions of EW-MSM total score (dependent variable) on various predictors.

Bold p<0.05.

### 3.3 Aim 1: clinicodemographic characteristics, baseline mood severity and current MDE treatment parameters as predictors of EW-MSM and categorical outcomes

Clinicodemographic characteristics, mood severity ratings and current MDE treatment characteristics of remitters across categorical outcomes are presented in **Supplementary Table 3** and **Table 2**, alongside results from univariate regressions of EW-MSM on the same predictors. Sex, age, and clinicodemographic characteristics and baseline mood ratings with *p* < 0.1 in the univariate analyses (comparisons and regressions), displayed in **Table 1**, were included as predictors of EW-MSM total score and categorical outcomes in multiple linear and multinomial logistic regression models, respectively (**Table 3**). Similar models were built with current MDE treatment characteristics with p<0.1 in univariate analyses (**Table 3**). Prior to model construction, collinearity tests were conducted, and the most informative predictors were retained. Lifetime and current episode psychotic features were collinear, and the latter was retained. R-MADRS and first evaluation MADRS were also collinear; the former was retained. Similarly, fluoxetine dose equivalents and number of AD trials were collinear with number of ADs used and were, therefore, excluded from multivariate models.

**Table 3:** Multiple linear regressions of EW-MSM total score and multinomial logistic regressions of categorical outcomes in relation to clinicodemographic characteristics, baseline mood severity and current MDE treatment characteristics in remitters (N = 233).

Predictors	EW- MSM total score		Categorical outcomes			
			2^nd^ AD trial remitters N=62		TRD remitters N=66	
			(reference: 1^st^ AD trial remitters)			
	B	*P*-value	RRR	*P*-value	RRR	*P*-value
Clinicodemographic characteristics and baseline mood severity						
Sex (male)	- 0.271	0.094	1.326	0.427	0.750	0.432
Age	- 0.002	0.756	1.010	0.465	0.994	0.665
Number of hospitalizations	0.018	0.718	–	–	–	–
Psychotic features in current episode	0.927	**<0.001**	–	–	–	–
OCD (lifetime)	0.763	**0.004**	3.774	**0.032**	4.198	**0.019**
First-degree relative with MDD	–	–	2.241	**0.040**	2.594	**0.013**
R-MADRS score	0.109	**<0.001**	1.169	**<0.001**	1.177	**<0.001**
	R^2^ = 0.330 Adj.R^2^ = 0.312		Cox-Snell pseudo-R^2^ = 0.200			
Current MDE treatment characteristics						
Current MDE duration (months)	0.032	**<0.001**	1.15	**0.001**	1.26	**<0.001**
Total number of ADs received	0.57	**<0.001**	-*	-*	-*	-*
AD combination in any trial	- 0.19	0.263	13.03	**0.004**	108.15	**<0.001**
Augmentation in any trial	1.56	**<0.001**	1.58	0.353	1.57	0.481
AD combination in last AD trial	-0.032	0.826	0.41	0.321	0.17	0.051
Augmentation in last trial	-**	-**	-**	-**	-**	-**
	R^2^ = 0.767 Adj.R^2^ = 0.762		Cox-Snell pseudo-R^2^ = 0.456			

Predictors included in the models were chosen based on univariate analyses (p < 0.1).

AD, Antidepressant; EW-MSM, Episode-wide Maudsley Staging Method; MDD, Major Depressive Disorder; MDE, Major Depressive Episode; OCD, Obsessive Compulsive Disorder; R-MADRS, Retrospective baseline Montgomery Åsberg Depression Rating Scale; RRR, Relative Risk Ratio; TRD, Treatment Resistant Depression.

*removed due to lack of convergence (predictor perfectly predicts outcome), **removed due to collinearity.

Bold p<0.05.

Clinicodemographic characteristics and baseline mood severity accounted for 33% of the variance of EW-MSM, but only current psychotic features, lifetime OCD comorbidity and R-MADRS emerged as significant predictors. Clinicodemographic characteristics and baseline mood severity explained 20% of the variance of categorical outcomes, but only lifetime OCD comorbidity, family history of MDD in first-degree relatives and R-MADRS were identified as significant predictors of both 2^nd^ AD trial remitters and TRD remitters relative to 1^st^ AD trial remitters.

Current MDE treatment characteristics accounted for 76.7% of the variance of EW-MSM, with current MDE duration, total number of ADs received and augmentation in any trial being significant predictors. Finally, current MDE treatment characteristics explained 45.6% of the variance of categorical outcomes, with current MDE duration and AD combination in any trial being significant predictors.

Therefore, EW-MSM and categorical outcomes were predicted by shared and distinct sets of clinicodemographic and treatment-related characteristics in multivariate models. Predictions were always more efficient for EW-MSM.

### 3.4 Aim 2: comparing the predictive value of EW-MSM versus categorical outcomes for improvement in depression severity

A hierarchical regression analysis was conducted to examine the comparative predictive value of EW-MSM total score and categorical outcomes for Δ-MADRS scores (**Table 4**). In the first model, age and sex accounted for 5.1% of the variance in Δ-MADRS scores (R²=0.051, *p* = 0.003). Adding categorical outcomes in the second model significantly improved model fit (ΔR²=0.066, *p* < 0.001). In the third model, EW-MSM total score was introduced to the first model, leading to a substantial increase in explained variance (ΔR²=0.143, *p* < 0.001). The final model included both categorical outcomes and EW-MSM apart from sex and age and accounted for 24.5% of the variance in Δ-MADRS scores (*p* < 0.001). Compared to model 2, EW-MSM total score improved R² by 0.127 (p<0.001) in the final model. Compared to model 3, categorical outcomes provided an additional ΔR² of only 0.051 (p<0.001) in the final model. These findings highlight that the EW-MSM staging tool captures MADRS improvement to remission more effectively compared to categorical outcomes.

**Table 4:** Hierarchical regression analysis of EW-MSM total score and categorical outcomes as predictors of Δ-MADRS scores in remitters (N = 233).

Model	Predictors	B	*P*-value	Model *p*-value	R^2^	ΔR^2^ (*p-*value)
**1**	Age	0.069	**0.023**	**0.003**	0.051	–
	Sex (male)	2.07	**0.008**			
**2**	Age	0.058	**0.046**	**<0.001**	0.117	Model 2-1: 0.066 (*p* < **0.001**)
	Sex (male)	1.81	**0.017**			
	Categorical outcomes (ref. 1^st^ AD trial remitters)					
	2^nd^ AD trial remitters	3.5	**<0.001**			
	TRD remitters	2.35	**0.007**			
**3**	Age	.057	**0.039**	**<0.001**	0.194	Model 3-1: 0.143 (*p* < **0.001**)
	Sex (male)	2.11	**0.003**			
	EW-MSM total score	1.57	**<0.001**			
**4**	Age	0.047	0.084	**<0.001**	0.245	Model 4-2: 0.127 (*p* < **0.001**) Model 4-3: 0.051 (*p* < **0.001**)
	Sex (male)	1.81	**0.010**			
	Categorical outcomes (ref. 1^st^ AD trial remitters)					
	2^nd^ AD trial remitters	1.94	**0.026**			
	TRD remitters	- 1.9	0.073			
	EW-MSM total score	1.95	**<0.001**			

AD, Antidepressant; EW-MSM, Episode-wide Maudsley Staging Method; MADRS, Montgomery-Åsberg Depression Rating Scale; TRD, Treatment Resistant Depression; Δ-MADRS, Retrospective baseline MADRS – Remission MADRS.

Bold p<0.05.

However, it should be noted that baseline depression severity (R-MADRS), embedded by definition in Δ-MADRS, is also included in EW-MSM as its severity dimension. As a result, R-MADRS was more strongly associated with EW-MSM (R^2^ = 0.241, p=1.55E-15) than with categorical outcomes (R^2^ = 0.137, p=4.18E-08), suggesting that EW-MSM superiority in predicting Δ-MADRS might be due to its stronger association with R-MADRS. This was formally tested by adjusting hierarchical regression models above for R-MADRS. Superiority of EW-MSM over categorical outcomes was lost after adjustment, corroborating our hypothesis.

### 3.5 Aim 3: discriminative utility of EW-MSM across categorical outcomes

**Figure 2:**
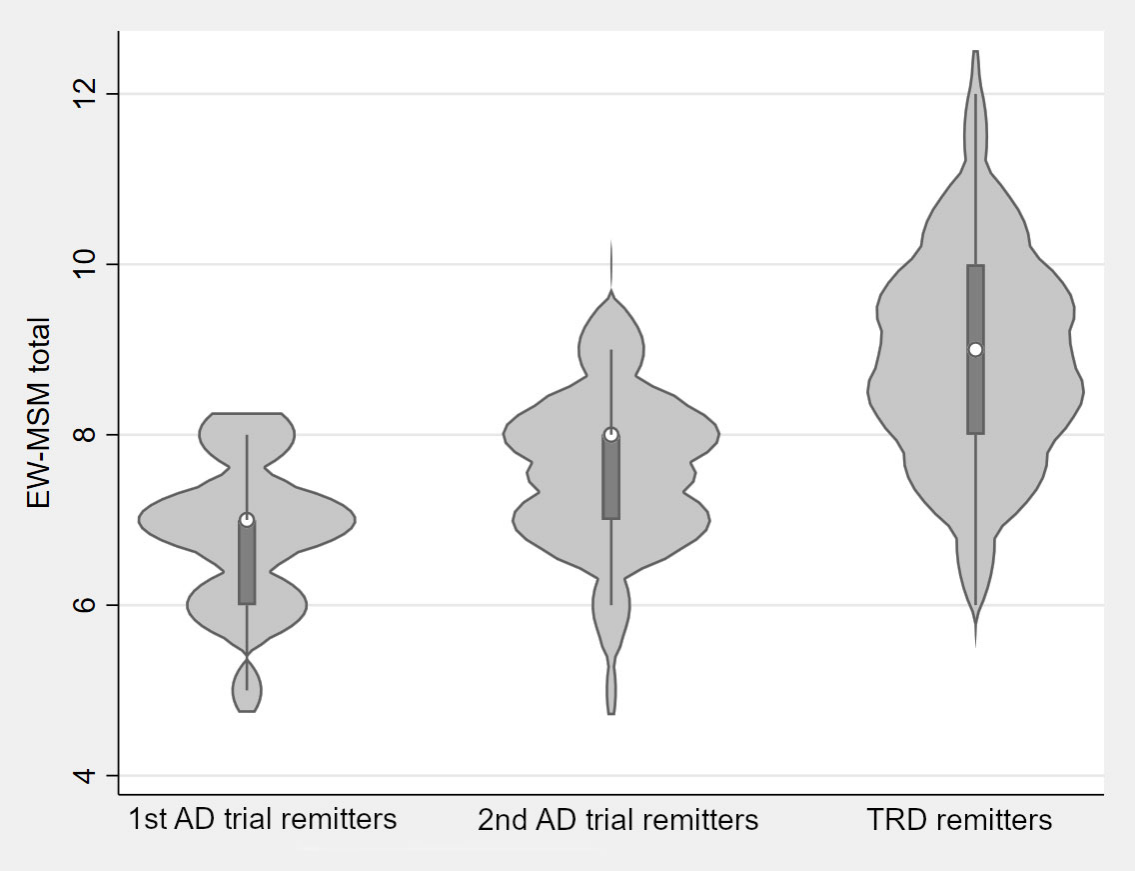
Violin plots of EW-MSM by remission-based categorical outcomes. AD, Antidepressant; EW-MSM, Episode-wide Maudsley Staging Method; TRD, Treatment-resistant depression.

**Table 5** presents descriptives for EW-MSM total score and dimensions, examined both as continuous and categorical variables, along with their comparisons across categorical outcomes. TRD remitters (group C1) exhibited the highest EW-MSM duration, analyzed either way (p<0.001). Although EW-MSM severity showed no significant differences when analyzed as a continuous variable, significant differences emerged when examined categorically (p=0.002). Moderate severity was more prevalent in group A, whereas severe depression without psychotic features was more prevalent in group C1 than A. No significant differences were observed for severe depression with psychotic features. EW-MSM Antidepressants was highest in group C1, lowest in group A and intermediate in group B (p<0.001; C1>B>A). Statistically significant differences were also found in EW-MSM Augmentation (p=0.009; C1>A). EW-MSM ECT scored 0 in all groups as no patient had received ECT treatment. EW-MSM total scores clearly distinguished between groups (p<0.001; C1>B>A) (**Figure 2**). Categorical outcomes explained 42% of the variance of EW-MSM (R^2^ = 0.42, *p* < 0.001) and vice versa. When established cut-offs for severity of resistance (27) were applied, 20.6% exhibited mild resistance, 76.8% moderate resistance and 2.6% severe resistance. Mild resistance was more frequent in group A, moderate resistance in groups B and C1 while severe resistance in C1 (p< 0.001). In summary, EW-MSM was strongly but not perfectly correlated with categorical outcomes; a clear gradient of resistance across all three outcome categories was displayed only by the Antidepressants dimension and by EW-MSM total score.

**Table 5:** Comparative analysis of EW-MSM dimensions as continuous and categorical variables across categorical outcomes in remitters (N = 233).

EW-MSM dimensions	Group A 1^st^ AD trial remitters N=105	Group B 2^nd^ AD trial remitters N=62	Group C1 TRD remitters N=66	All remitters N=233	Comparison between groups A, B, C1	
							Statistic	*p*-value
EW-MSM Duration (1-3) (cont.)	1 (1, 1)	1 (1, 1)	1.5 (1, 2)	1 (1, 1)	56.882^1^	**<0.001** **(C1>A=B)**
EW-MSM Duration (cat.)					54.546^2^	**<.001**
Acute (≤ 12 months)	101 (96.2%)	53 (85.5%)	33 (50%)	187 (80.3%)		**(A>B>C1)**
Sub-acute (13–24 months)	4 (3.8%)	6 (9.7%)	20 (30.3%)	30 (12.9%)		**(C1>A=B)**
Chronic (> 24 months)	0 (0%)	3 (4.8%)	13 (19.7%)	16 (6.9%)		**(C1>A=B)**
EW-MSM Severity (1-5) (cont.)	4 (3, 4)	4 (4, 4)	4 (4, 4)	4 (4, 4)	4.764^1^	0.092
EW-MSM Severity (cat.)					16.747^3^	**0.002**
Moderate	27 (25.7%)	5 (8.1%)	4 (6.1%)	36 (15.5%)		**(A>B=C1)**
Severe without psychosis	57 (54.3%)	45 (72.6%)	50 (75.8%)	152 (65.2%)		**(C1>A)**
Severe with psychosis	21 (20%)	12 (19.4%)	12 (18.2%)	45 (19.3%)		**(A=B=C1)**
EW-MSM Antidepressants (1-5) (cont.)	1 (1, 1)	1 (1, 2)	2 (2, 2)	1 (1, 2)	147.847^1^	**<0.001** **(C1>B>A)**
EW-MSM Antidepressants (cat.)					177.336^2^	**<0.001**
Level 1: 1–2 Antidepressants	105 (100%)	39 (62.9%)	6 (9.1%)	150 (64.4%)		**(A>B>C1)**
Level 2: 3–4 Antidepressants	0 (0%)	23 (37.1%)	47 (71.2%)	70 (30%)		**(C1>B>A)**
Level 3: 5–6 Antidepressants	0 (0%)	0 (0%)	9 (13.6%)	9 (3.9%)		**(C1>A=B)**
Level 4: 7–10 Antidepressants	0 (0%)	0 (0%)	4 (6.1%)	4 (1.7%)		**(C1>A)**
EW-MSM Augmentation (cat.)	79 (75.2%)	54 (87.1%)	61 (92.4%)	194 (83.3%)	9.480^3^	**0.009** **(C1>A)**
EW-MSM ECT (cat.)	0 (0%)	0 (0%)	0 (0%)	0 (0%)	–	–
EW-MSM total score (3-15) (cont.)	7 (6, 7)	8 (7, 8)	9 (8, 10)	7 (7, 8)	96.995^1^	**<0.001** **(C1>B>A)**
EW-MSM total score (cat.)					44.340^2^	**<0.001**
Mild resistance (3–6 points)	39 (37.1%)	7 (11.3%)	2 (3%)	48 (20.6%)		**(A>B=C1)**
Moderate resistance (7–10 points)	66 (62.9%)	55 (88.7%)	58 (87.9%)	179 (76.8%)		**(B=C1>A)**
Severe resistance (11–15 points)	0 (0%)	0 (0%)	6 (9.1%)	6 (2.6%)		**(C1>A=B)**

Displayed are median (25th, 75th percentiles) or N (%) in first 4 columns.

AD, Antidepressant; cat., categorical; cont., continuous; ECT, Electroconvulsive Therapy; EW-MSM, Episode-wide Maudsley Staging Method; TRD, Treatment Resistant Depression

^1^ Kruskall-Wallis test, ^2^ Fisher-Freeman-Halton exact test, ^3^ Chi-square test

Bold p<0.05

**Figure 3:**
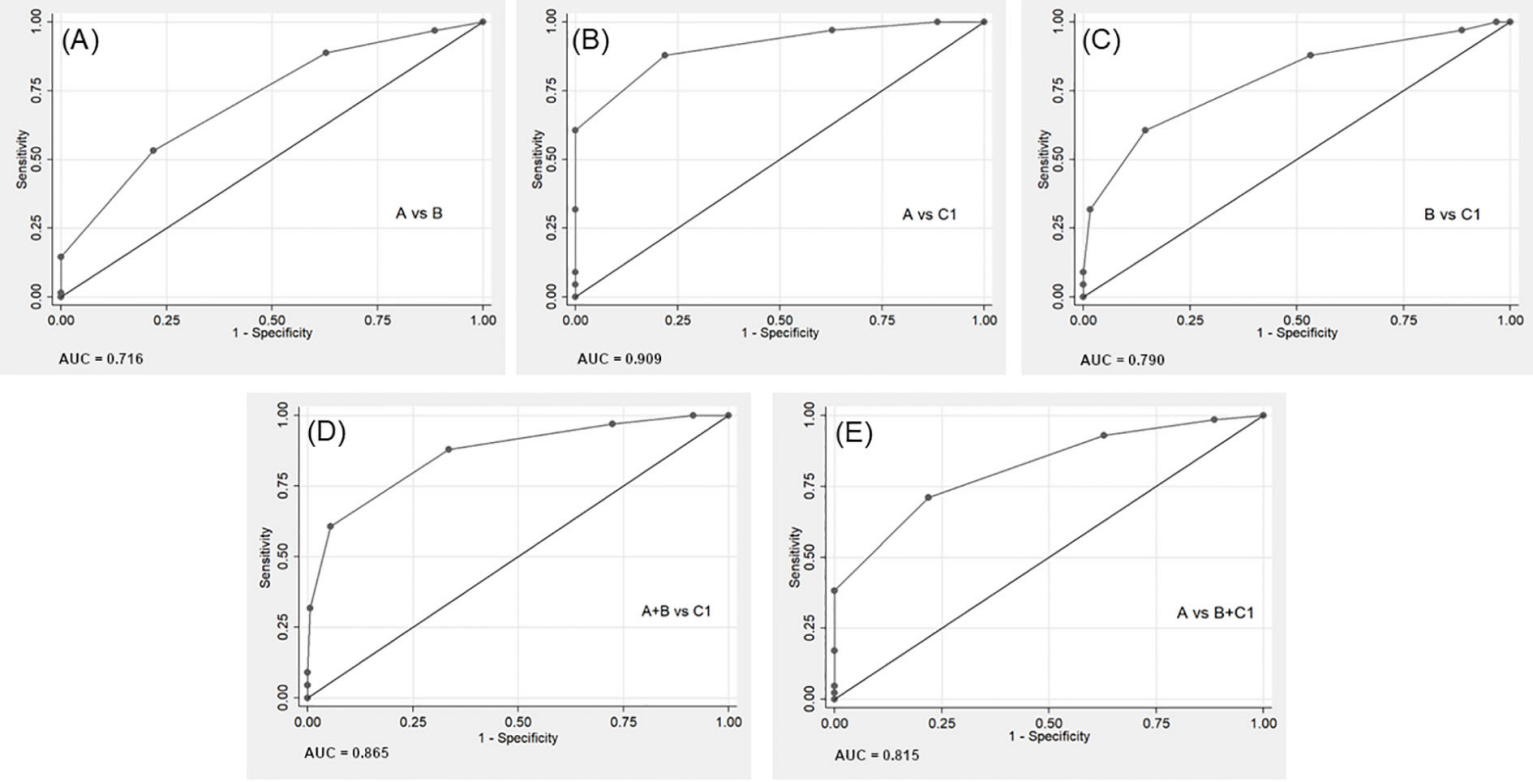
Comparative ROC Curve analyses for remission-based categorical outcomes using EW-MSM total score as classifier. **(A)** Comparison between group A vs. group B: AUC = 0.716; optimal cut-off=7/8: Sn=53.23%, Sp=78.10%, PPV = 58.93%, NPV = 73.87%, PLR = 2.43, NLR = 0.60, Acc=68.86%, BAC = 65.67%. **(B)** Comparison between group A vs. group C1: AUC = 0.909; optimal cut-off=7/8: Sn=87.88%, Sp=78.1%, PPV = 71.6%, NPV = 91.11%, PLR = 4.01, NLR = 0.155, Acc=81.87%, BAC = 82.99%. **(C)** Comparison between group B vs. C1: AUC = 0.79; optimal cut-off=8/9: Sn=60.61%, Sp=85.48%, PPV = 81.63%, NPV = 67.09%, PLR = 4.18, NLR = 0.46, Acc=72.66%, BAC = 73.05%. **(D)** Comparison between combined groups A and B vs. C1: AUC = 0.865; optimal cut-off=8/9: Sn=60.61%, Sp=94.61%, PPV = 81.63%, NPV = 85.87%, PLR = 11.25, NLR = 0.42, Acc=84.98%, BAC = 77.61%. **(E)** Comparison between group A vs. combined groups B and C1: AUC = 0.815; optimal cut-off=7/8: Sn=71.09%, Sp=78.1%, PPV = 79.82%, NPV = 68.91%, PLR = 3.25, NLR = 0.37, Acc=74.25%, BAC = 74.60%. Group definitions: group A = 1^st^ AD trial remitters; group B = 2^nd^ AD trial remitters; group C1 = TRD remitters; Acc, Accuracy; AD, Antidepressant; AUC, Area Under the Curve; BAC, Balanced Accuracy; EW-MSM, Episode-wide Maudsley Staging Method; NLR, Negative Likelihood Ratio; NPV, Negative Predictive Value; ROC Curve, Receiver Operating Characteristic Curve; PLR, Positive Likelihood Ratio; PPV, Positive Predictive Value; Sn, sensitivity; Sp, specificity; TRD, Treatment-resistant depression.

ROC curve analyses were conducted to evaluate the discriminative utility of EW-MSM total scores for categorical outcomes (**Figure 3**). AUC values, sensitivity, specificity, accuracy (Acc, i.e., correct classification rates) and balanced accuracy (BAC) were calculated for various comparisons. The highest classification accuracy was observed in the comparison of the most distant categories A vs. C1 (AUC = 0.909, 95% CI = 0.86-0.95; Acc=81.9%, BAC = 83.0% at a cut-off score of 7/8). Comparison of the two last categories (B vs. C1, AUC = 0.790, 95% CI = 0.72-0.86) yielded an Acc=72.7% and BAC = 73% at the more rigorous cut-off of 8/9. Distinction between A and B was poor (AUC = 0.716, 95% CI = 0.64-0.79; Acc=68.9%, BAC = 65.7% at a cut-off of 7/8). Comparisons were also performed for combined groups. Comparison between A and the two last groups combined (A vs. B+C1) exhibited AUC = 0.815 (95% CI = 0.76-0.87) and Acc=74.3%, BAC = 74.6% at a cut-off of 7/8. Comparison between the two first groups combined and C1 (A+B vs. C1) exhibited AUC = 0.865 (95% CI = 0.81-0.92) and Acc=85.0%, BAC = 77.6% at a cut-off of 8/9. These findings suggest that EW-MSM total score effectively (AUC≥0.80) discriminated between the most distant categories (1^st^ AD trial remitters vs. TRD remitters) and between either of these vs. all other categories combined but not among adjacent categories. The most efficient and reliable discrimination (AUC 95% CI beyond 0.80) was between TRD remitters and 1^st^ AD trial remitters or 1^st^ and 2^nd^ AD trial remitters combined. However, these findings should be interpreted cautiously as the two measures partially share treatment exposure information (number of ADs in EW-MSM and number of AD trials in categorical outcomes, which are highly correlated), which can inflate discrimination performance.

## 4 Discussion

This study is, to our knowledge, the first to apply EW-MSM, a dimensional score of treatment resistance recorded over the whole depressive episode, from episode onset to remission, as previous research only employed MSM at randomly timed snapshots, such as at hospital discharge, to predict short- or long-term outcomes. This score summarizes most of the clinical and treatment-related information that is required for treatment resistance quantification and that was collected during our naturalistic trial. It was, therefore, considered as a gold standard against which typical remission-based categorical outcomes were compared. Comparison between these two measures of resistance focused on three targets: 1) which were their clinical and treatment-related predictors, 2) how well the two measures predicted depression improvement to remission, and 3) how the two measures were related with each other.

The prevalence of TRD using our remission-based categorical definition was 37.5% (100 TRD patients out of 267 in total). This compares to around 45% of patients cumulatively failing to achieve remission at the second step of the STAR*D study, in which treatment options were more limited, available only after the first step and sequenced in a structured way (20). Using their response-based definition, the GSRD recorded a TRD prevalence of 50.7% and 40.6% in the TRD-I (47) and TRD-III (45, 48, 49) cross-sectional studies, respectively, and 54.5% in the TRD-II (50) prospective study. In summary, discrepances across cohorts may be attributed to different recruitment strategies, settings, treatment procedures followed and TRD definitions employed.

EW-MSM and categorical outcomes were predicted by shared and distinct sets of clinicodemographic variables in multivariate models. Baseline depressive severity and lifetime OCD comorbidity emerged as significant predictors for both EW-MSM and categorical models, while current psychotic features were associated with EW-MSM outcomes only, and family history of MDD in first-degree relatives was significant only in the categorical framework. Baseline depression severity was more strongly associated with EW-MSM than with categorical outcomes, since it is reflected by its severity dimension. Baseline depression severity also emerged as a key predictor of resistance in STAR*D findings, showing that higher initial symptom scores were associated with reduced likelihood of remission at the second step (51), and in GSRD results (25), that similarly identified severity as a robust correlate of resistance in TRD-I (47), TRD-II (52) and TRD-III (53) studies. Of note, mean R-MADRS was 40.6 in our tertiary-care study, with >80% of remitters being hospitalized during their current MDE, while in TRD-II and TRD-III studies mean R-MADRS was 31.5 and 34.1, respectively (48, 50). Lifetime OCD comorbidity (in 9.4% of remitters in our study) was also identified as predictor of resistance at the STAR*D second step (51); in the TRD-III study, the prevalence of comorbid OCD was very low (1.65%) and, although linked with higher suicidal risk and polypharmacy, it was not associated with resistance (54). Current psychotic features (in 19.3% of remitters), a predictor embedded within the MSM severity dimension, were consequently strongly linked with EW-MSM scores in our cohort, in agreement with the TRD-III study (53), where prevalence of psychotic features was 10.9% (48). The association between family history of MDD (in 28.4% of remitters) and categorical outcomes corroborates similar findings in the TRD-II study (52) pointing to the heritable vulnerability of TRD (55). GSRD studies have identified additional clinical predictors of resistance, including number of hospitalizations, suicidality, recurrent episodes, early age of onset, comorbid anxiety disorders and anhedonia/melancholic features (47, 48, 52, 53, 56–58). These factors were not replicated in our study, which may be resulting from our remission-based definition as opposed to response-based frameworks, as well as the single tertiary-care center design and limited power of our sample.

In our study, remitters received a median of 2 ADs per episode, rising to 4 among TRD remitters. Nearly half (49.4%) received AD combinations and 83.3% underwent augmentation during their current MDE; respective percentages in TRD remitters were 84.8% and 92.4%. These rates indicate substantial treatment intensity and are broadly consistent with findings from the GSRD studies. In TRD-I, 18.7% of TRD patients had three and 9.2% four ADs during the index episode (47). In TRD-III, 60.6% received combination or augmentation regimens (45), with 29.5% on AD combinations and augmentation often involving antipsychotics (25.7%) or mood stabilizers (11.3%), while mean number of psychiatric drugs administered reached 2.18. Our cohort showed high pharmacological exposure, with mean cumulative fluoxetine equivalent doses of 89.9 mg across all trials versus 39.9 mg in TRD-III (45). This difference likely reflects the longer follow-up of our study subjects followed up to remission, demanding more AD trials and more intensive dose optimization. Treatment-related characteristics were unsurprisingly strongly associated with both EW-MSM and categorical outcomes. Longer episode duration predicted higher resistance in both EW-MSM and categorical models. Moreover, in multivariate models EW-MSM was associated with the total number of ADs received while categorical outcomes with the use of AD combinations. Additionally, EW-MSM was significantly predicted by the use of augmentation strategies, since these are included as a separate indicator of resistance. Overall, both clinicodemographic and treatment-related characteristics predicted EW-MSM more efficiently than categorical outcomes, underscoring its superior ability to reflect the multidimensional aspects of resistance (clinical burden, treatment intensity).

The hierarchical regression analysis demonstrated the superiority of EW-MSM over categorical outcomes in capturing symptom improvement from baseline to remission. This was mainly attributed, in a sensitivity analysis, to the fact that EW-MSM, through its severity dimension, was more strongly associated with baseline depression severity than categorical outcomes. Furthermore, EW-MSM can better reflect several other key aspects of resistance while categorical outcomes only rely on discrete AD trial counts. As a result, EW-MSM provides a more accurate and comprehensive index of resistance, which also captures therapeutic gain more efficiently than categorical outcomes.

Comparative analyses of EW-MSM dimensions across categorical outcomes showed a clear gradient of resistance, increasing from 1^st^ to 2^nd^ AD trial and TRD remitters (C1>B>A), only for the Antidepressants dimension, as number of ADs in EW-MSM is highly correlated with number of (monotherapy or combination) AD trials in categorical outcomes. Other EW-MSM dimensions were not equally discriminative. EW-MSM Duration clearly separated TRD remitters from early remitters but not among the two early remitters subgroups. EW-MSM Severity performed poorly and only when examined categorically while EW-MSM Augmentation clearly separated only among the most distant categories. These findings suggest that typical categorical outcomes are clearly separated among them only by the number of ADs received, based on which they are defined, but less clearly by several other key aspects of resistance, such as clinical severity, episode duration, and augmentation strategies. Yet, EW-MSM total score was strongly though not perfectly correlated with categorical outcomes and performed as well as the Antidepressants dimension, clearly distinguishing between all three categories on the same gradient, maybe because this dimension contributes up to 5 points to the total score.

The ROC curve analyses demonstrated that EW-MSM discriminated effectively (AUC≥0.80) only between the most distant categories (1^st^ AD trial remitters vs. TRD remitters; AUC = 0.91) but not among adjacent categories. The suboptimal discriminative efficiency of EW-MSM between adjacent categories, especially among the first two, arises from the fact that our sample included patients with high severity intensely treated early on. Even in 1^st^ AD trial remitters, 20% received AD combination and 75% received augmentation. Most remitters (76.8%) had moderate resistance by MSM cut-offs, making it more difficult to distinguish subgroups in them. As noted above, number of ADs tightly aligned with outcome categories, but other EW-MSM dimensions showed higher dispersion across them, resulting in poor discrimination between adjacent categories in ROC analyses. As has been proposed, MSM could become more granular and increase its discriminative efficiency by incorporating the number of augmentation strategies and AD combinations used (13).

Comparisons in ROC analyses were also extended to include combined groups. As pharmacological trials in TRD patients are sparse (15, 59), treatment guidance often relies on trials in patients failing to remit after at least one adequate AD trial (60). Psychiatric pharmacogenetic studies also often focus on non-remitters after one AD trial (61). Therefore, comparing 1^st^ AD trial remitters with all other groups is of clinical relevance; discrimination among them was fair in our study (AUC = 0.815). When 1^st^ and 2^nd^ AD trial remitters were combined and contrasted with TRD remitters, the discriminative efficiency was even higher (AUC = 0.865), suggesting that TRD remitters are quite distinct from early remitters and lending validity to the TRD definition. Discriminative cut-offs identified in ROC analyses included EW-MSM scores of 8 and 9 or higher. The former is useful in distinguishing 1^st^ AD trial remitters from all other groups combined or TRD remitters; the latter in distinguishing between TRD and early remitters. Both cut-offs lie in the moderate resistance range of scores (27).

Overall, findings from the comparisons between EW-MSM and categorical outcomes on our three aims are summarized as follows: 1) EW-MSM score was significantly associated with both clinicodemographic and treatment-related predictors, overlapping with, but also extending beyond those associated with categorical outcomes. Both approaches converged on baseline severity, current MDE duration, lifetime OCD comorbidity and number of ADs or AD combination as significant predictors in multivariate models, while EW-MSM was additionally predicted by current psychotic features and use of augmentation strategies. However, predictions were always more efficient for EW-MSM than for categorical outcomes, demonstrating its superior ability to reflect the clinical burden and treatment intensity aspects of resistance. 2) EW-MSM was more strongly associated with baseline severity than categorical outcomes and consequently predicted depression improvement to remission more efficiently. 3) EW-MSM was strongly though not perfectly associated with categorical outcomes. Only the Antidepressants dimension and the total score showed a clear gradient of resistance across all three outcome categories. As a result, EW-MSM effectively discriminated between the most distant categories (1^st^ AD trial remitters vs. TRD remitters) but not among adjacent categories. This does not necessarily point to an EW-MSM drawback; categorical outcomes defined by number of AD trial failures were not as distinct regarding other key aspects of resistance captured by EW-MSM and were therefore not adequately distinguishable by it.

This study has notable strengths. To our knowledge, this is the first application of MSM across the entire episode, whereas previous research had employed it at random snapshots, such as at hospital discharge (28–32). We derived a continuous score of episode-wide resistance, capturing both clinical burden (symptom severity, episode duration) and treatment intensity (AD trials, AD combination and augmentation). This was used as a reference against which remission-based categorical classifications were compared. The longitudinal, naturalistic design of the study mirrors real-world clinical challenges related to following up TRD patients to remission. We prioritized clinical remission over symptom response, aligning with contemporary treatment objectives emphasizing remission and functional recovery beyond remission, but recognizing that among them clinical remission is the most feasible and finally the preferred endpoint to define a successful clinical outcome (22).

Several limitations should also be acknowledged. First, the cohort did not include patients receiving ECT or newer interventions indicated in TRD, such as esketamine, which may limit generalizability to all therapeutic strategies. Moreover, unavailability of ECT did not allow scoring of the full EW-MSM tool in this cohort. Total scores might be higher, particularly for TRD patients, if ECT was available. Second, MSM does not capture non-pharmacological interventions, like psychotherapy or rTMS; consequently, patients receiving psychotherapy were excluded from our study to maintain consistency in applying MSM, though at the cost of external validity. Third, no objective measures of adherence, such as AD plasma levels, were used; suboptimal adherence may lead to pseudo-resistance (12). Fourth, the timing of episode onset and baseline depression severity were retrospectively determined and, therefore, potentially subject to recall bias. Finally, this study was conducted at a single tertiary-care center and included intensely treated patients with high severity, introducing selection bias; therefore, replication in other clinical settings is needed to ensure the findings are representative of broader clinical populations.

Our findings suggest several directions for future clinical and translational research. Following up MDD (incl. TRD) patients to remission in clinical research settings and using EW-MSM to measure treatment resistance could help identify data-driven cut-offs of resistance through latent class/profile or cluster analyses of EW-MSM dimensions, possibly combined with patient-reported outcomes. These cut-offs could prove of greater clinical utility than typical categorical classifications and be used for drug discovery or stratification in clinical trials. Furthermore, EW-MSM total score or specific dimensions might be tested, in an unbiased and hypothesis-free way (i.e. without relying on arbitrary cut-offs based on AD trial failures), for correlation with putative biological (e.g. inflammatory or oxidative stress biomarkers, neuroimaging data, polygenic scores, etc.), clinical (e.g. psychiatric or medical comorbidity) or psychological (e.g. personality profile, cognition, childhood trauma, coping strategies, etc.) predictors. Timing should be considered in these analyses, facilitating the identification of stage-specific biomarkers for use in precision psychiatry frameworks. In such pragmatic studies follow-up can be long to accurately reflect routine care but could be justified by the expected benefits. Future studies should evaluate EW-MSM scoring in larger and more diverse populations to confirm applicability and strengthen generalizability. Finally, feasibility could be enhanced by applying the EW-MSM approach in electronic health records databases or patient registries.

In conclusion, our study highlighted the clinical importance of prioritizing remission over response as the therapeutic target and for defining resistance, showing that remission is feasible even among patients with TRD, yet after more treatment attempts. EW-MSM produced a continuous score of episode-wide resistance, capturing both clinical burden (symptom severity, episode duration) and treatment intensity, which was used as a reference against which remission-based categorical outcomes were compared. EW-MSM and categorical outcomes were predicted by shared but also distinct sets of clinicodemographic and treatment-related variables. However, EW-MSM reflected clinical burden, incl. baseline depression severity, and treatment intensity aspects of resistance more strongly than categorical outcomes and consequently predicted depression improvement to remission more efficiently. EW-MSM scores followed a clear gradient of resistance across categorical outcomes and effectively discriminated between the most distant categories but not among adjacent ones. Overall, our results demonstrate the added value of using EW-MSM and support its use in clinical and translational research to identify the clinical, biological and psychological correlates of resistance.

## Data Availability

The raw data supporting the conclusions of this article will be made available by the authors, without undue reservation.
