# Supplementary material for: Episode-wide Maudsley staging in treatment-resistant depression: a longitudinal tertiary-care comparison of dimensional and categorical outcomes
Source: Front Psychiatry. 2026 Feb 11;17:1769246. doi: 10.3389/fpsyt.2026.1769246 (PMC12932473; doi:10.3389/fpsyt.2026.1769246)
Supplement: Supplementary file 1 [file Table1.docx]

**Supplementary Table 1.** Descriptive statistics and comparisons of clinicodemographic data and mood ratings between TRD remitters and TRD non-remitters (N=100).

| **Variables** | **Group C1**  **TRD remitters**  **N=66** | **Group C2**  **TRD non-remitters**  **N= 34** | **Comparison C1 vs. C2** | |
| --- | --- | --- | --- | --- |
|  |  |  | **Statistic** | ***p*-value** |
| **Clinicodemographic characteristics** |  |  |  |  |
| Age | 51.95 ± 12.98 | 56.91 ± 10.1 | -1.242^1^ | 0.214 |
| Sex (female) | 44 (66.7%) | 25 (73.5%) | 0.494^2^ | 0.482 |
| Education (years) | 12 (9, 16) | 12 (6, 16) | 0.685^1^ | 0.493 |
| Employment |  |  | 1.040^2^ | .594 |
| Employed | 18 (27.3%) | 9 (26.5%) |  |  |
| Unemployed/student | 29 (43.9%) | 12 (35.5%) |  |  |
| Retired | 26 (28.8%) | 13 (38.2%) |  |  |
| Family status |  |  | 4.941^2^ | 0.085 |
| Single | 12 (18.2%) | 5 (14.7%) |  |  |
| Married/Cohabiting | 39 (59.1%) | 14 (41.2%) |  |  |
| Divorced/Widow | 15 (22.7%) | 15 (44.1%) |  |  |
| Living alone | 12 (18.2%) | 9 (26.5%) | 0.929^2^ | 0.335 |
| Recurrent MDD | 49 (74.2%) | 26 (76.5%) | 0.059^2^ | 0.807 |
| Number of MDEs | 2 (1, 3) | 2 (1.75, 4) | - 0.564^1^ | 0.572 |
| Age of onset (years) | 41.3 ± 15.1 | 43.29 ± 16.1 | - 0.586^1^ | 0.558 |
| History of suicide attempts (lifetime) | 32 (48.5%) | 19 (55.9%) | 0.491^2^ | 0.483 |
| Number of suicide attempts | 0 (0, 1) | 1 (0, 2.25) | - 1.266^1^ | 0.206 |
| Number of hospitalizations | 1 (1, 2) | 1.5 (1, 2.25) | - 0.667^1^ | 0.505 |
| Psychotic features (lifetime) | 15 (22.7%) | 7 (20.6%) | 0.060^2^ | 0.807 |
| Current psychotic features | 12 (18.2%) | 5 (14.7%) | 0.192^2^ | 0.661 |
| Atypical depressive features (lifetime) | 10 (15.2%) | 5 (14.7%) | 0.003^2^ | 0.953 |
| Melancholic depressive features (lifetime) | 41 (62.1%) | 24 (70.6%) | 0.707^2^ | 0.400 |
| Anxiety disorders (lifetime) | 26 (39.4%) | 14 (41.2%) | 0.030^2^ | 0.863 |
| OCD (lifetime) | 9 (13.6%) | 4 (11.8%) | -^3^ | 1 |
| Dysthymia (lifetime) | 11 (16.7%) | 2 (5.9%) | -^3^ | 0.209 |
| AUD (lifetime) | 10 (15.2%) | 4 (11.8%) | -^3^ | 0.767 |
| First-degree relative with MDD | 23 (35.4%) | 10 (29.4%) | 0.358^2^ | 0.549 |
| First-degree relative with BD | 2 (3.1%) | 2 (5.9%) | -^3^ | 0.605 |
| First-degree relative with schizophrenia | 5 (7.7%) | 3 (8.8%) | -^3^ | 1 |
| **Mood ratings: baseline/ final (remission or study end)** |  |  |  |  |
| R-MADRS score | 42.44 ± 5.4 | 43.44 ± 6.1 | - 0.918^1^ | 0.358 |
| First evaluation MADRS score | 41.24 ± 5.05 | 40.94 ± 6.15 | 0.055^1^ | 0.956 |
| R-YMRS score | 1 (0, 2) | 1 (0, 2) | 0.149^1^ | 0.882 |
| Final MADRS score | 6.7 ± 2.36 | 22.82 ± 6.13 | - 8.211^1^ | **<0.001** |
| Final YMRS score | 0 (0, 0) | 0 (0, 1) | - 2.662^1^ | **0.008** |
| Δ-MADRS score | 35.74 ± 5.98 | 20.62 ± 6.63 | 7.327^1^ | **<0.001** |

Displayed are mean±SD or median (25th, 75th percentiles) or N (%) in first 2 columns.

AUD = Alcohol Use Disorder; BD = Bipolar Disorder; MADRS = Montgomery Åsberg Depression Rating Scale; MDD = Major Depressive Disorder; MDE = Major Depressive Episode; OCD = Obsessive Compulsive Disorder; R-MADRS = Retrospective baseline MADRS; R-YMRS = Retrospective baseline YMRS; TRD = Treatment Resistant Depression; YMRS = Young Mania Rating Scale; Δ-MADRS = Retrospective baseline MADRS – final MADRS

^1^ Mann-Whitney U test (standardized test statistic), ^2^ Chi-square test, ^3^ Fisher’s exact test

Bold p<0.05

**Supplementary Table 2.** Descriptive statistics and comparisons of current MDE treatment characteristics between TRD remitters and TRD non-remitters (N=100)

| **Current MDE treatment characteristics** | **Group C1**  **TRD remitters**  **N=66** | **Group C2=C-C1**  **TRD non-remitters**  **N= 34** | **Comparison C1 vs. C2** | |
| --- | --- | --- | --- | --- |
|  |  |  | **Statistic** | ***p*-value** |
| Current MDE duration (months) | 13 (8, 21) | 20 (14, 38.5) | - 2.889^1^ | **0.004** |
| Total number of ADs received | 4 (3, 4) | 3.5 (2, 5) | 0.786^1^ | 0.432 |
| Total number of AD trials | 3 (3, 4) | 3 (2, 4) | 1.503^1^ | 0.133 |
| AD combination in any trial | 56 (84.8%) | 22 (64.7%) | 5.306^2^ | **0.021** |
| Fluoxetine dose equivalents for all ADs across MDE, mg/day | 147.5 ± 59.83 | 162.72 ± 79.9 | - 0.749^1^ | 0.454 |
| Augmentation in any trial | 61 (92.4%) | 31 (91.2%) | -^3^ | 1 |
| AD combination in first trial | 10 (15.2%) | 8 (23.5%) | 1.067^2^ | 0.302 |
| AD combination in last AD trial | 36 (54.5%) | 14 (41.2%) | 1.604^2^ | 0.205 |
| Fluoxetine dose equivalents for ADs in last trial, mg/day | 67.35 ± 29.7 | 69.54 ± 36.92 | 0.007^1^ | 0.994 |
| Augmentation in last trial | 57 (86.4%) | 28 (82.4%) | 0.283^2^ | 0.595 |

Displayed are mean±SD or median (25th, 75th percentiles) or N (%) in first 2 columns.

AD = Antidepressant; MDE = Major Depressive Episode; TRD = Treatment Resistant Depression

^1^ Mann-Whitney U test (standardized test statistic), ^2^ Chi-square test, ^3^ Fisher’s exact test

Bold p<0.05

**Supplementary Table 3.** Descriptive statistics and univariate analyses of clinicodemographic data and mood ratings in remitters (N=233).

| **Variables** | **Group A**  **1^st^ AD trial remitters**  **N=105** | **Group B**  **2^nd^ AD trial remitters**  **N=62** | **Group C1**  **TRD remitters**  **N=66** | **All remitters**  **N=233** | **Comparison between groups A, B, C1** | | **EW-MSM on predictors*** | |
| --- | --- | --- | --- | --- | --- | --- | --- | --- |
|  |  |  |  |  | **Statistic** | ***p*-value** | **B** | ***p*-value** |
| **Clinicodemographic characteristics** |  |  |  |  |  |  |  |  |
| Age | 51.37 ± 12.75 | 54.23 ± 11.48 | 51.95 ± 12.98 | 52.3 ± 12.5 | 2.712^1^ | 0.258 | 0.007 | 0.336 |
| Sex (female) | 69 (65.7%) | 33 (53.2%) | 44 (66.7%) | 146 (62.7%) | 3.230^2^ | 0.199 | 0.024 | 0.901 |
| Education (years) | 12 (8, 16) | 12 (9, 14.5) | 12 (9, 16) | 12 (8, 16) | 1.073^1^ | 0.585 | - 0.011 | 0.630 |
| Employment |  |  |  |  | 1.019^2^ | 0.907 |  |  |
| Employed | 29 (27.6%) | 21 (33.9%) | 18 (27.3%) | 68 (29.2%) |  |  | (reference) | (reference) |
| Unemployed/student | 48 (45.7%) | 26 (41.9%) | 29 (43.9%) | 103 (44.2%) |  |  | 0.029 | 0.895 |
| Retired | 28 (26.7%) | 15 (24.2%) | 26 (28.8%) | 62 (26.6%) |  |  | 0.147 | 0.553 |
| Family status |  |  |  |  | 1.134^2^ | 0.889 |  |  |
| Single | 20 (19%) | 14 (22.6%) | 12 (18.2%) | 46 (19.7%) |  |  | (reference) | (reference) |
| Married/Cohabiting | 58 (55.2%) | 31 (50%) | 39 (59.1%) | 128 (54.9%) |  |  | 0.234 | 0.334 |
| Divorced/Widow | 27 (25.7%) | 17 (27.4%) | 15 (22.7%) | 59 (25.3%) |  |  | 0.185 | 0.504 |
| Living alone | 27 (25.7%) | 14 (22.6%) | 12 (18.2%) | 53 (22.7%) | 1.310^2^ | 0.519 | - 0.318 | 0.147 |
| Recurrent MDD | 75 (71.4%) | 46 (74.2%) | 49 (74.2%) | 170 (73%) | 0.228^2^ | 0.892 | - 0.137 | 0.508 |
| Number of MDEs | 2 (1, 4) | 3 (1, 4) | 2 (1, 3) | 2 (1, 4) | 2.445^1^ | 0.294 | - 0.037 | 0.270 |
| Age of onset (years) | 40.75 ± 14.88 | 39.5 ± 13.7 | 41.3 ± 15.1 | 40.58 ± 14.6 | 0.616^1^ | 0.735 | 0.008 | 0.194 |
| History of suicide attempts (lifetime) | 59 (56.2%) | 29 (46.8%) | 32 (48.5%) | 120 (51.5%) | 1.719^2^ | 0.423 | 0.052 | 0.778 |
| Number of suicide attempts | 1 (0, 2) | 0 (0, 2) | 0 (0, 1) | 1 (0, 2) | 1.483^1^ | 0.476 | 0.024 | 0.646 |
| Number of hospitalizations (lifetime) | 1 (1, 2) | 1 (1, 2) | 1 (1, 2) | 1 (1, 2) | 3.673^1^ | 0.159 | 0.121 | **0.037** |
| Psychotic features (lifetime) | 27 (25.7%) | 13 (21%) | 15 (22.7%) | 55 (23.6%) | 0.526^2^ | 0.769 | 0.996 | **<0.001** |
| Current psychotic features | 21 (20%) | 12 (19.4%) | 12 (18.2%) | 45 (19.3%) | 0.086^2^ | 0.958 | 1.226 | **<0.001** |
| Atypical depressive features (lifetime) | 14 (13.3%) | 5 (8.1%) | 10 (15.2%) | 29 (12.4%) | 1.611^2^ | 0.447 | - 0.371 | 0.182 |
| Melancholic depressive features (lifetime) | 62 (59%) | 46 (74.2%) | 41 (62.1%) | 149 (63.9%) | 4.012^2^ | 0.135 | 0.197 | 0.303 |
| Anxiety disorders (lifetime) | 31 (29.5%) | 15 (24.2%) | 26 (39.4%) | 72 (30.9%) | 3.629^2^ | 0.163 | 0.024 | 0.903 |
| OCD (lifetime) | 5 (4.8%) | 8 (12.9%) | 9 (13.6%) | 22 (9.4%) | 4.916^2^ | 0.086 | 0.579 | 0.065 |
| Dysthymia (lifetime) | 12 (11.4%) | 9 (14.5%) | 11 (16.7%) | 32 (13.7%) | 0.982^2^ | 0.612 | - 0.077 | 0.773 |
| AUD (lifetime) | 17 (16.2%) | 11 (17.7%) | 10 (15.2%) | 38 (16.3%) | 0.159^2^ | 0.924 | 0.361 | 0.147 |
| First-degree relative with MDD | 22 (21%) | 21 (33.9%) | 23 (35.4%)** | 66 (28.4%)** | 5.330^2^ | 0.070 | 0.291 | 0.155 |
| First-degree relative with BD | 4 (3.8%) | 6 (9.7%) | 2 (3.1%) | 12 (5.2%) | 3.106^3^ | 0.190 | - 0.068 | 0.870 |
| First-degree relative with schizophrenia | 7 (6.7%) | 4 (6.5%) | 5 (7.7%) | 16 (6.9%) | 0.186^3^ | 0.947 | 0.199 | 0.585 |
| **Mood ratings: baseline/ remission** |  |  |  |  |  |  |  |  |
| R-MADRS score | 38.3 ± 5.23 | 42.63 ± 5.39 | 42.44 ± 5.4 | 40.62 ± 5.7 | 32.013^1^ | **<0.001**  **(B=C1>A)** | 0.121 | **<0.001** |
| First evaluation MADRS score | 37.4 ± 5 | 41.73 ± 5.18 | 41.24 ± 5.05 | 39.64 ± 5.44 | 33.772^1^ | **<0.001**  **(B=C1>A)** | 0.125 | **<0.001** |
| R-YMRS score | 1 (0, 2) | 2 (.075, 2) | 1 (0, 2) | 1 (0, 2) | 3.600^1^ | 0.165 | 0.086 | 0.324 |
| Remission MADRS score | 4.91 ± 2.37 | 5.35 ± 2.5 | 6.7 ± 2.36 | 5.54 ± 2.51 | 22.182^1^ | **<0.001**  **(C1>A=B)** | **-** | **-** |
| Remission YMRS score | 0 (0, 0) | 0 (0, 0) | 0 (0, 0) | 0 (0, 0) | 9.506^1^ | **0.009**  **(B>A)** | - | - |
| Δ-MADRS score | 33.38 ± 5.19 | 37.27 ± 5.86 | 35.74 ± 5.98 | 35.09 ± 5.82 | 20.748^1^ | **<0.001**  **(B=C1>A)** | - | - |

Displayed are mean±SD or median (25^th^, 75^th^ percentiles) or N (%) in first 4 columns.

AUD = Alcohol Use Disorder; BD = Bipolar Disorder; EW-MSM = Episode-wide Maudsley Staging Method; MADRS = Montgomery Åsberg Depression Rating Scale; MDD = Major Depressive Disorder; MDE = Major Depressive Episode; OCD = Obsessive Compulsive Disorder; R-MADRS = Retrospective baseline MADRS; R-YMRS = Retrospective baseline YMRS; TRD = Treatment Resistant Depression; YMRS = Young Mania Rating Scale; Δ-MADRS = Retrospective baseline MADRS – Remission MADRS

^1^  Kruskall-Wallis test , ^2^ Chi-square test, ^3^ Fisher-Freeman-Halton exact test

* Univariate linear regressions of EW-MSM total score (dependent variable) on various predictors, ** Missing data, total N=232.

Bold p<0.05
